# Identification and Validation of cGAS‐STING Pathway‐Associated Predictive and Therapeutic Models for Esophageal Squamous Cell Cancer Patients via Artificial Intelligence and Multi‐Omics

**DOI:** 10.1002/cam4.71645

**Published:** 2026-02-23

**Authors:** Chunyang Zhou, Xiaoli Liu, Zijian Wang, Tao Yang

**Affiliations:** ^1^ Department of Radiotherapy, Qilu Hospital of Shandong University (Qingdao) Shandong University Qingdao Shandong China; ^2^ Qilu Hospital of Shandong University (Qingdao) Qingdao Shandong China

**Keywords:** cGAS‐STING pathway, drug design, esophageal squamous cell cancer, molecular docking, prognostic model

## Abstract

**Background:**

Esophageal squamous cell cancer (ESCC) is a malignancy derived from the Esophagus, and dysregulation of the cGAS‐STING pathway contributes to ESCC progression.

**Method:**

ESCC bulk‐seq dataset GSE38129 was acquired from GEO database and then underwent Limma and WGCNA analysis for the identification of shared DEGs, which were intersected with cGAS‐STING pathway gene list and underwent Cox regression analysis for recognized cGAS‐STING associated prognostic indicators. Next, Consensus clustering and machine learning combinations (Lasso + SurvivalSVM) were utilized for cGAS‐STING associated ESCC molecular subgroups and prognostic model construction in TCGA‐ESCC cohorts, and prognostic performance was validated in GSE53662. Besides, hub prognostic variables were acquired from Lasso‐Cox regression, and their molecular and immune features were estimated via multiple bioinformatic approaches at TCGA‐ESCC cohort. In addition, heterogeneity of hub genes at single‐cell level for ESCC patients was also indicated in GSE188900 in spatial and temporal manners. Furthermore, Drug sensitivity and molecular docking analysis were performed for identification of optimal therapeutic agents targeting hub genes. Indeed, in vitro assays have been performed to assess the oncogenic potential of hub genes and efficacy of optimal therapeutic agents. Furthermore, implications of hub genes with cGAS‐STING pathway were estimated in single‐cell artificial intelligence (AI) driven‐virtual cell and bulk assays.

**Results:**

By utilizing integrative AI and multi‐omic pipelines, we proved that the cGAS‐STING pathway can guide subgroup stratification and prognostic model construction for ESCC patients. PRKDC and SLC25A13 can be considered hub genes associated with ESCC pathogenesis and regulation of the cGAS‐STING pathway. BX‐912 and Navitoclax can be considered drug screening strategies for the treatment of ESCC patients by targeting PRKDC and SLC25A13.

**Conclusion:**

cGAS‐STING pathway can guide risk stratification and can be considered as a therapeutic target for ESCC patients, which provides novel insights into precision and personalized medicine for ESCC patients.

## 1 Introduction

Esophageal squamous cell carcinoma (ESCC) is a malignant neoplasm originating from the esophagus, with approximately 500,000 new cases reported globally each year, resulting in a mortality rate as high as 50% [1, 2]. The incidence of ESCC is particularly elevated in Asia, especially in China, where environmental and genetic factors contribute to its pathogenesis [3, 4]. Current treatment options, including surgery, radiotherapy, and chemotherapy, have shown limited efficacy, highlighting the urgent need for novel therapeutic strategies [5, 6]. The cGAS‐STING pathway, an innate immune response regulator, has been implicated in the reprogramming of the tumor microenvironment (TME) of ESCC, thereby facilitating ESCC tumor progression and radiotherapy and chemotherapy resistance [7, 8]. Hence, deeper insights into the cGAS‐STING pathway in ESCC pathogenesis can provide novel ideas for ESCC clinical translation. In recent years, Artificial intelligence (AI) approaches, such as machine learning and deep learning algorithms, facilitate the development of prognostic biomarkers and therapeutic agents for cancer patients [9]. In ESCC, numerous reports discovered novel predictive models and therapeutic screening frameworks for ESCC patients [10, 11, 12]. However, it has not yet been elucidated whether the cGAS‐STING pathway can be considered a target for elaborating predictive and druggable potentials.

**FIGURE 1:**
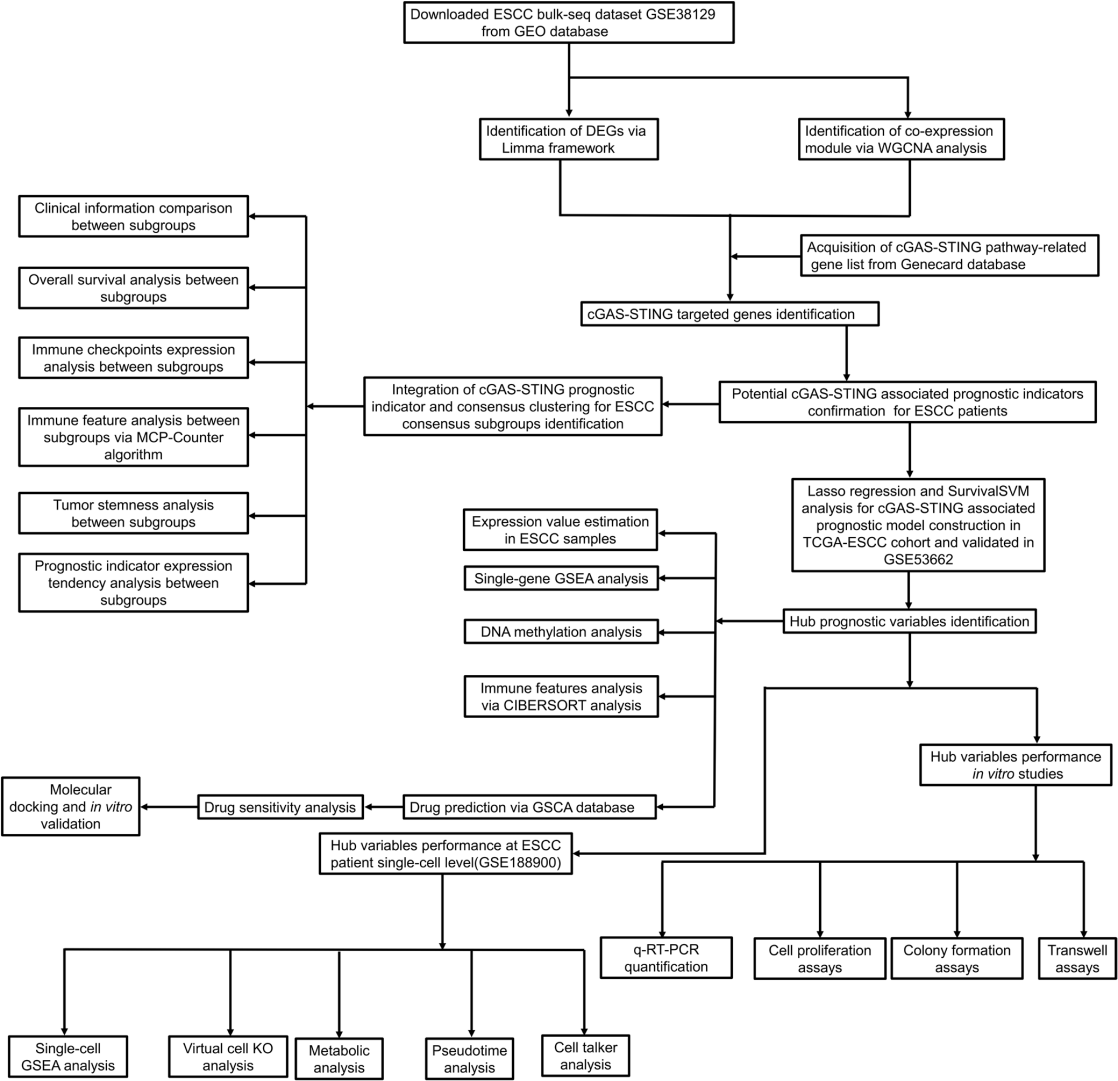
The workflow of this study.

In this study, we utilized public transcriptomic datasets for elucidating cGAS‐STING pathway mechanisms at bulk and single‐cell level. Integrated AI and multi‐omics results indicated that ESCC patients can be divided into 2 molecular and risk groups based on cGAS‐STING pathway, and PRKDC and SLC25A13 can be considered as 2 hub genes potential regulating cGAS‐STING pathway. Besides, Drug sensitivity and molecular docking results speculated that ESCC patients may be potentially benefited from BX‐912 and Navitoclax treatment. Furthermore, in vitro assays proved that RKDC and SLC25A13 tumor‐promoting potential and BX‐912 and Navitoclax therapeutic potentials for ESCC patients. Overall, our study first illustrated cGAS‐STING pathway predictive and therapeutic potentials for ESCC patients, which enhance ESCC clinical translation. We illustrated the workflow of this study in Figure 1.

## 2 Material and Methods

### 2.1 Bulk and Pathological Data Source

ESCC bulk profiles, such as GSE38129 (based on GPL571, including 30 tumors and adjacent normal tissue) and GSE53662 (based on GPL18109, including 119 tumors and adjacent normal tissue) were downloaded from GEO database via GEOquery packages of R [13]. Besides, we downloaded STAR‐counts data and corresponding clinical information for ESCC tumors from the TCGA database. We then extracted data in TPM format and performed normalization using the log2 (TPM + 1) transformation. After retaining samples that included both RNAseq data and clinical information, we ultimately selected 83 tumors and 1 adjacent normal tissue sample for further analysis [14]. cGAS‐STING pathway‐related gene list was downloaded from Genecard database with the threshold > 1. Aforementioned datasets were subjected to normalization and standardization via Limma package of R software [15]. Immunohistochemistry (IHC) samples of ESCC patients were downloaded from the human protein atlas (HPA) database (HPA035174 for PRKDC in ESCC patient sample, CAB005167 for PRKDC in para‐cancer sample of ESCC patient, HPA018997 for SLC25A13 in ESCC patient sample, and HPA018997 for SLC25A13 in para‐cancer sample of ESCC patient).

### 2.2 Identification of Differentially Expressed Genes(DEGs)

After pre‐processing using R software limma package, DEGs were identified in GSE38129, with the following criterion: *p* < 0.05 and |log fold change (FC)| > 0.5 [15]. The results were visualized by a volcano map generated by the ggplot2 package of R software [16]. GSEA analysis was performed to evaluate DEGs' molecular functions in accordance with the hallmark gene set downloaded from the MSIGDB database via the clusterProfiler package of R software [17].

### 2.3 WGCNA Analysis

In the present investigation, we employed the “WGCNA” package within the R programming environment to explore the relationship between genes and phenotypic traits through the construction of a gene co‐expression network [18]. Initially, we excluded 50% of the genes that exhibited the lowest median absolute deviation (MAD) [18]. Subsequently, we computed Pearson's correlation matrices for all gene pairs and established a weighted adjacency matrix by applying the average linkage approach alongside a weighted correlation coefficient. The “soft” thresholding power (β) was utilized to derive the adjacency, which was subsequently transformed into a topological overlap matrix (TOM) [18]. To categorize genes exhibiting analogous expression patterns into distinct modules, we conducted average linkage hierarchical clustering based on the dissimilarity measure derived from TOM, setting a minimum threshold for gene group size at 50. Ultimately, we assessed the dissimilarity among module eigengenes, identified an appropriate cut line for the module dendrogram, and amalgamated several modules. WGCNA was utilized to pinpoint significant modules in ESCC, culminating in the development of a visualized eigengene network. Indeed, the cGAS‐STING associated gene list was intersected with Limma and WGCNA results for the identification of cGAS‐STING associated DEGs.

### 2.4 Potential Prognostic Factors Evaluation

For confirming the prognostic indicators among cGAS‐STING associated DEGs, we performed univariate cox regression via the survival package of R. Friend analysis of cGAS‐STING associated prognostic indicators was analyzed via the GOSemSim package of R software. PPI network of cGAS‐STING associated prognostic indicators was enriched by the STRING database and illustrated by Cytoscape software. Besides, the expression patterns of cGAS‐STING associated prognostic indicators with various clinical parameters were analyzed by a heatmap generated by ComplexHeatmap of R software. ne Ontology (GO) provides a commonly applied framework for gene function annotation, encompassing the three categories of Molecular Function (MF), Biological Process (BP), and Cellular Component (CC). In addition, KEGG enrichment analysis serves as an effective approach for exploring gene activities and associated high‐level genomic pathways. For a more comprehensive understanding of the oncogenic mechanisms of these target cGAS‐STING associated indicators, the ClusterProfiler package in R was utilized to perform GO term analysis and to identify significantly enriched KEGG pathways [17].

### 2.5 Consensus Clustering

For classifying cGAS‐STING associated molecular subgroups, we performed consensus clustering. The ConsensusClusterPlus package within the R software environment was employed to classify ESCC patients into distinct consensus subtypes in TCGA‐ESCC cohort [19]. During each iteration, 80% of the samples were randomly selected, and clustering was performed using Pearson correlation distance. This clustering procedure was executed repeatedly for ten iterations, culminating in the aggregation of results from each iteration to establish a stable classification. Additionally, tumor stemness was assessed using the OCLR algorithm within the R framework. To gain further insight into the implications of precision medicine within cGAS‐STING associated subgroups, immune infiltration and immune checkpoint expression levels were analyzed and visualized employing the ssGSEA algorithms with ggplot2 and pheatmap packages of R software, respectively. Besides, clinical parameters between subgroups were also analyzed. The expression patterns of cGAS‐STING associated prognostic indicators were also analyzed.

### 2.6 Machine Learning and Prognostic Model Construction

To establish a prognostic signature associated with the cGAS‐STING pathway, we devised a comprehensive workflow that integrated ten distinct machine learning methodologies [20]. These included Random Survival Forest (RSF), Elastic Network (Enet), Lasso regression, Ridge regression, stepwise Cox regression, CoxBoost, Partial Least Squares Regression for survival (plsRcox), Supervised Principal Components (SuperPC), Generalized Boosted Regression Modeling (GBM), and Survival Support Vector Machine (survival‐SVM) [20]. We evaluated a total of 101 unique model configurations based on cGAS‐STING pathway‐related candidate prognostic factors pertinent to patients with ESCC [20]. The training of the signature was conducted using the TCGA‐ESCC cohort, while its effectiveness was subsequently validated using an independent dataset (GSE53662) to predict the overall survival (OS) risk for ESCC patients through a combination of Lasso and SurvivalSVM configuration [21]. The analytical framework encompassed the aforementioned 10 methodologies [21]. To enhance the reliability of our findings, we implemented both leave‐one‐out cross‐validation (LOOCV) and 10‐fold cross‐validation techniques, which aided in minimizing errors across the various model combinations [21]. For each model configuration, the concordance index (C‐index) was computed utilizing both the TCGA‐ESCC dataset and the GSE53662 dataset [22]. The configuration with the highest C‐index value across these datasets was identified as optimal, facilitating the selection of a specific model for the particular study. Following the identification of the optimal model, we evaluated the performance of the prognostic model for OS prediction in ESCC patients across both training and independent validation datasets using Kaplan–Meier (KM) curve analysis and time‐dependent Receiver Operating Characteristic (ROC) curve analysis utilizing R software. Furthermore, to determine the accuracy of the prognostic model for ESCC patients within the TCGA‐ESCC cohort, we conducted nomogram and calibration analysis, using the survival, survminer, timeROC, rms, and ggplot2 packages in R software. In order to develop cGAS‐STING associated significant variables, we utilized Lasso‐Cox regression technique within the TCGA‐ESCC cohort. via survival and rms packages of R software.

### 2.7 Molecular and Immune Features Analysis of Hub Genes

Based on the TCGA‐ESCC cohort, we compared the expression value of targeted genes and their functions via single‐gene GSEA analysis produced in R software. Next, the relationships between the DNA methylation level of targeted genes and clinical outcomes of ESCC patients were performed in the GSCA database. Besides, the immune features of targeted genes were performed by the CIBERSORT algorithm in R software. Besides, the co‐expression correlation between cGAS‐STING regulators with cGAS‐STING pathways and hub genes was also assessed in the TCGA‐ESCC cohort, and their corresponding hub genes with the cGAS‐STING interaction network were estimated by the GeneMANIA database [23]. The expression of hub genes in histological level was performed by human protein atlas(HPA) database.

### 2.8 Single‐Cell Transcriptomic Analysis

To initiate our investigation, we obtained the single‐cell transcriptomic dataset related to ESCC (GSE188900, including 7 ESCC tissue samples) from the GEO repository. The analysis of the single‐cell RNA sequencing (scRNA‐seq) data involved several essential steps, such as quality control (QC), dimensionality reduction, and identification of markers, all conducted using the Seurat R package [24]. We implemented QC measures for each individual cell, following specific criteria which mandated gene counts to fall within the range of 200 to 6000, a unique molecular identifier (UMI) count to exceed 1000, and the percentage of mitochondrial genes to be maintained below 10% [24]. After the QC processes, the data were normalized, which enabled the identification of 2000 genes with significant variability for subsequent analysis [24]. Following normalization, we employed dimensionality reduction techniques, specifically t‐SNE and UMAP [24]. Cell type annotations were performed utilizing the singleR algorithm within the R environment [25]. Intercellular communication networks were inferred using the Celltalk package in R [26]. Moreover, the investigation of energy metabolic pathways at the single‐cell level among the annotated cell populations was conducted through the scMetabolism package in R [27]. Additionally, pseudo‐time analysis of targeted gene expression within specific cell types was performed using the monocle2 package in R [28]. ScTenifoldKnk was utilized for the identification of knockout (KO) of hub genes in targeted cells [29]. We also executed single‐cell gene set enrichment analysis (ssGSEA) to explore the functional enrichment of the hub gene at a single‐cell resolution, in alignment with the hallmark gene set sourced from the MsigDB database via ClusterProfiler package of R [17].

### 2.9 Drug Prediction and Validation by Molecular Docking

We utilized the GSCA (Gene Set Cancer Analysis) database to analyze target‐drug sensitivity, aiming to predict the effects of various drugs on the expression of the targeted gene. Besides, the Oncopredict package was utilized for the prediction of drug sensitivity to ESCC [30]. For molecular docking, the X‐ray crystal structure of targeted genes was retrieved from the RCSB PDB (Research Collaboratory for Structural Bioinformatics Protein Data Bank) database. The 3D molecular structure of Lapatinib was obtained from the Pubchem database. Autodock vina and PyMOL software were utilized for molecular docking, and a binding energy threshold of < −5.0 kcal/mol indicates favorable binding potential [31]. The results of molecular docking were visualized using PyMOL software [31]. BX‐912 (HY‐11005) and Navitoclax (HY‐10087) were purchased from MCE (China).

### 2.10 Cell Line, Culture and Conditions

The KYSE150, HET1, and HEK 293 T cell lines were acquired from the Shanghai Academy of Biological Sciences located in Shanghai, China. The KYSE150 and HET1 were cultured using Roswell Park Memorial Institute (RPMI) 1640 complete medium, supplemented with a 1% antibiotic mixture of penicillin–streptomycin and 10% fetal bovine serum (FBS, Gibco). In contrast, the HEK 293 T cell line was maintained in Dulbecco's Modified Eagle Medium (DMEM), also enriched with a 1% penicillin–streptomycin mixture and 10% FBS. All cell passage procedures were conducted under controlled conditions of 37°C, 5% CO2, within a humidified incubator.

### 2.11 RNA Extraction and q‐RT‐PCR

Total RNA was isolated utilizing TRIzol reagent (TaKaRa, Beijing, China), and its concentration, purity, and integrity were assessed with a NanoDrop spectrophotometer (Thermo Scientific, Waltham, MA, USA). For reverse transcription, 1 μg of total RNA was utilized alongside HiScript II Q RT SuperMix for qPCR (+gDNA wiper) and a gDNA eraser (Vazyme, Shanghai, China). The concentration, purity, and integrity of the resultant cDNA were subsequently evaluated using the aforementioned NanoDrop spectrophotometer. Quantitative reverse transcription polymerase chain reaction (qRT‐PCR) was performed employing SYBR Green MasterMix (11203ES50, YEASEN, Shanghai, China) and StepOne Software v.2.3 (Applied Biosystems, Carlsbad, CA, USA) across 40 cycles, with three biological replicates for each sample. Data analysis was conducted using the ∆∆Ct (cycle threshold) methodology and normalized against the expression levels of the reference gene, GAPDH. The primer sequences utilized in the qRT‐PCR assays are as follows:

SLC25A13.

F 5′–GTGGAACTTTTAAGTGGAGTGGT–3′.

R 5′–TTGATGAATTGTGGTCTGTCCAA–3′.

PRKDC.

F: 5′–ATTCTTTGTCGGGAGCAGCA–3′.

R: 3′–CCTAGCTGTGTGGCACATGA–5′.

GAPDH.

F 5′–GAGAAGGCTGGGGCTCATTT–3′.

R 5′–ATGACGAACATGGGGGCATC–3′.

### 2.12 Silencing via shRNA

The target sequences for silencing PRKDC and SLC25A13 were as follows:

shRNA for PRKDC: GCGACATATTATGGAAGAATT.

shRNA for SLC25A13: GCCATCTACTTTCCGTGCTAT.

All sequences were integrated into the pLKO.1 lentiviral plasmid. In essence, the plasmids responsible for encoding shRNA were co‐transfected alongside the VSV‐G envelope plasmid and the psPAX packaging plasmid into HEK293T cells. This transfection utilized Lipofectamine 2000 (Thermo Fisher Scientific), in accordance with the manufacturer's guidelines. The growth medium was changed the subsequent day, and the supernatants containing lentivirus were collected 3 days post‐transfection, filtered, and then used to infect target cells in the presence of 4 μg/mL polybrene (Sigma‐Aldrich). Subsequently, the KYSE150 cell line was cultured at a density of 5 × 10^4 cells per well in 24‐well plates until a confluence of 50%–70% was attained. Following this, the standard culture medium was substituted with a diluted sh‐SLC25A13 and sh‐PRKDC lentiviral stock solution for transfection purposes. After a 72‐h incubation period, the cells underwent trypsin digestion and were rinsed with phosphate‐buffered saline (PBS). The cells were subsequently plated onto 10 cm culture dishes at a density of 500 cells per dish, and puromycin (Thermo Scientific) selection was carried out for a duration of 3 weeks. Surviving clones were identified by the appearance of cloning rings, after which they were expanded and subcloned utilizing the limiting dilution method.

### 2.13 Cell Proliferation Assays

Cells in the logarithmic phase of growth were subjected to trypsinization, followed by enumeration and subsequent plating into 96‐well plates at a concentration of 3000 cells per well (*n* = 6). Following incubation at predetermined intervals (4, 24, 48, 72, 96, and 120 h), 10 μL of CCK‐8 reagent was introduced to each well, and the plates were incubated for an additional 2 h. Absorbance readings were taken at 450 nm using a microplate reader. The cell proliferation rate was determined using the formula (A_d − A_blank_d)/(A_4h − A_blank_4h). All experiments were conducted in triplicate to ensure reliability of the results. For IC50 validation, cells were seeded in 96‐well plates at a density of 2000 cells per well (100 μL per well) and cultured for 24 h (at 37°C, 5% CO_2_) following drug treatment. Subsequently, 10 μL of Cell Counting Kit‐8 (Cell Counting Kit‐8, C0038, Beyotime) reagent was added to each well, taking care to avoid bubble formation. The plates were then returned to the incubator for 2 h. The absorbance was measured at 450 nm using a microplate reader (EnSight, PerkinElmer, USA), and the cell survival rate was calculated according to the manufacturer's protocol.

### 2.14 Wound Healing Assays

A wound‐healing assay was conducted to evaluate cellular migration post‐treatment. KYSE150 control cells, sh‐PRKDC KYSE150 cell lines, and sh‐SLC25A13 cell lines were seeded at 3 × 10^5^ cells per well in 6‐well plates and incubated overnight. Wounds were created. Wound closure was monitored at 0 and 24 h post‐treatment using an inverted microscope, and closure rates were analyzed with ImageJ. The results were derived from three independent sets (*n* = 3) of triplicate experiments.

### 2.15 Transwell Assays

The experiment employed Transwell chambers produced by Corning Inc., based in New York, USA. Each chamber received 150 μL of a cell suspension, containing 10,000 cells. In the lower compartment, 600 μL of culture medium enriched with 10% fetal bovine serum was added. After a 24‐h incubation period, non‐migratory cells were removed, and the migrated cells were stained with a 0.25% crystal violet solution for 10 min. Following this, the chambers were washed with phosphate‐buffered saline (PBS) and subsequently captured in images. For the in vitro cell invasion assay, Transwell chambers that had been pre‐coated with a matrix gel at a concentration of 100 micrograms per square centimeter, sourced from Thermo Fisher Scientific, were utilized.

### 2.16 Western Blotting(WB)

After administering various treatments, the cells were rinsed with ice‐cold phosphate‐buffered saline (PBS) (Hyclone, Seattle, WA, USA) and subsequently harvested via gentle scraping. Total protein extraction was achieved by lysing the cells with radioimmunoprecipitation assay (RIPA) lysis buffer (Beyotime, Shanghai, China), which was augmented with a mixture of phosphatase inhibitors (Beyotime, China) and protease inhibitors (Beyotime, China). The resulting cell lysates were subjected to centrifugation at 14,000 × g for 15 min at 4°C. Following this, the lysates were denatured for 10 min in a 5× SDS‐PAGE loading buffer (Beyotime, China). The proteins were then separated via SDS‐PAGE and subsequently transferred onto polyvinylidene fluoride (PVDF) membranes (Beyotime, China) for Western blotting analysis. The membranes were blocked using NcmBlot blocking buffer (NCM Biotech, Suzhou, China) for 10 min. They were incubated with primary antibodies for 8 h at 4°C and then diluted in 5% bovine serum albumin (BSA) (Solarbio, Beijing, China). Following this, the membranes were treated with secondary antibodies (ThermoFisher, Waltham, MA, USA), which were diluted in WB secondary antibody diluent solution (Beyotime, Shanghai, China) at a dilution ratio of 1:1000 for 2 h at room temperature. Protein detection was performed using an enhanced chemiluminescence (ECL) substrate (Thermo Fisher, Waltham, MA, USA). The quantification of protein expression was carried out by analyzing the band densities of the target proteins using ImageJ software version 1.57, with the analysis based on the density values relative to the GAPDH protein. The primary antibody used in this study was illustrated as follows: PRKDC (ab32566, ABCAM, USA: 1:2000), SLC25A13 (ab167166, ABCAM, USA: 1:2000), GAPDH (No. 66009–1‐Ig, Proteintech Group Inc., CHINA: 1:1000).

### 2.17 Statistical Analysis

For bioinformatic analysis, all statistical evaluations were conducted utilizing R software (version 4.2.2). The Wilcoxon test was employed to assess the differences in the proportions of immune‐infiltrated cells present within the tumor microenvironment. To examine the relationships among different variables, Pearson correlation analysis was applied. A *p*‐value or FDR of less than 0.05 was considered statistically significant. Data are presented as mean ± SD, *p < 0.05, **p < 0.01, ***p < 0.001. For the experimental part, all statistical evaluations were conducted utilizing GraphPad Prism (Version 8.0.2); all experiments were conducted with a minimum of three biological replicates, and the results are presented as the mean ± standard deviation (SD). The statistical analysis to evaluate the differences between the two data sets was performed using either two‐way ANOVA or Student's *t*‐test, with a *p*‐value of less than 0.05 deemed statistically significant.

## 3 Results

### 3.1 Identification of cGAS‐STING Associated DEGs for ESCC Patients

**FIGURE 2:**
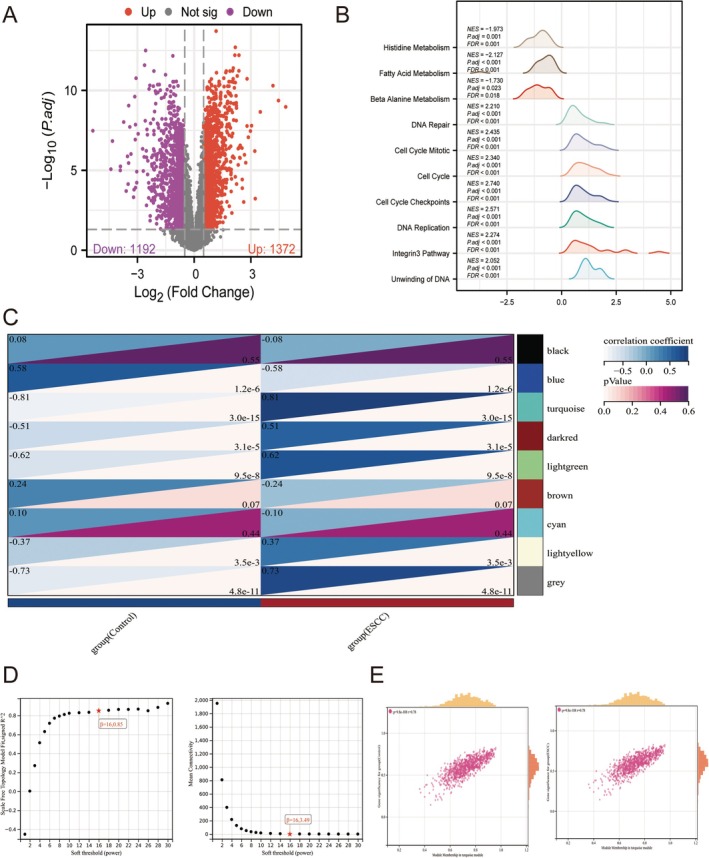
WGCNA and Limma analysis in GSE38129 dataset. (A) Volcano plot showing DEGs between ESCC patients and healthy controls in internal set. (B) GSEA analysis of DEGs in GSE38129 (C) Module trait relationship heatmap generated by WGCNA in GSE38129. (D) Scatterplots of representative modules in GSE38129. (E) Correlation module of WGCNA in GSE38129. Data with threshold of *p* < 0.05 and was considered as significant.

In ESCC bulk profile GSE38129, we first underwent normalization and standardization (Figure S2A,B). Next, we identified 1372 up‐regulated and 1192 down‐regulated DEGs (Figure 2A). Besides, DEGs in GSE38129 mainly involved in regulating energy metabolism, DNA repair, and cell cycle (Figure 2B). Besides, we performed WGCNA analysis for the identification of co‐expression model in GSE38129 and discovered that turquoise model (correlation co‐efficient = −0.81 and *p* = 3.0e‐15 in normal samples, and correlation co‐efficient = 0.81 and *p* = 3.0e‐15 in ESCC samples) was the optimal module with the β threshold = 16.085 and 16.349 (Figures 2C–E and S1C–E).

### 3.2 cGAS‐STING Associated Prognostic Indicators Identification for ESCC Patients

**FIGURE 3:**
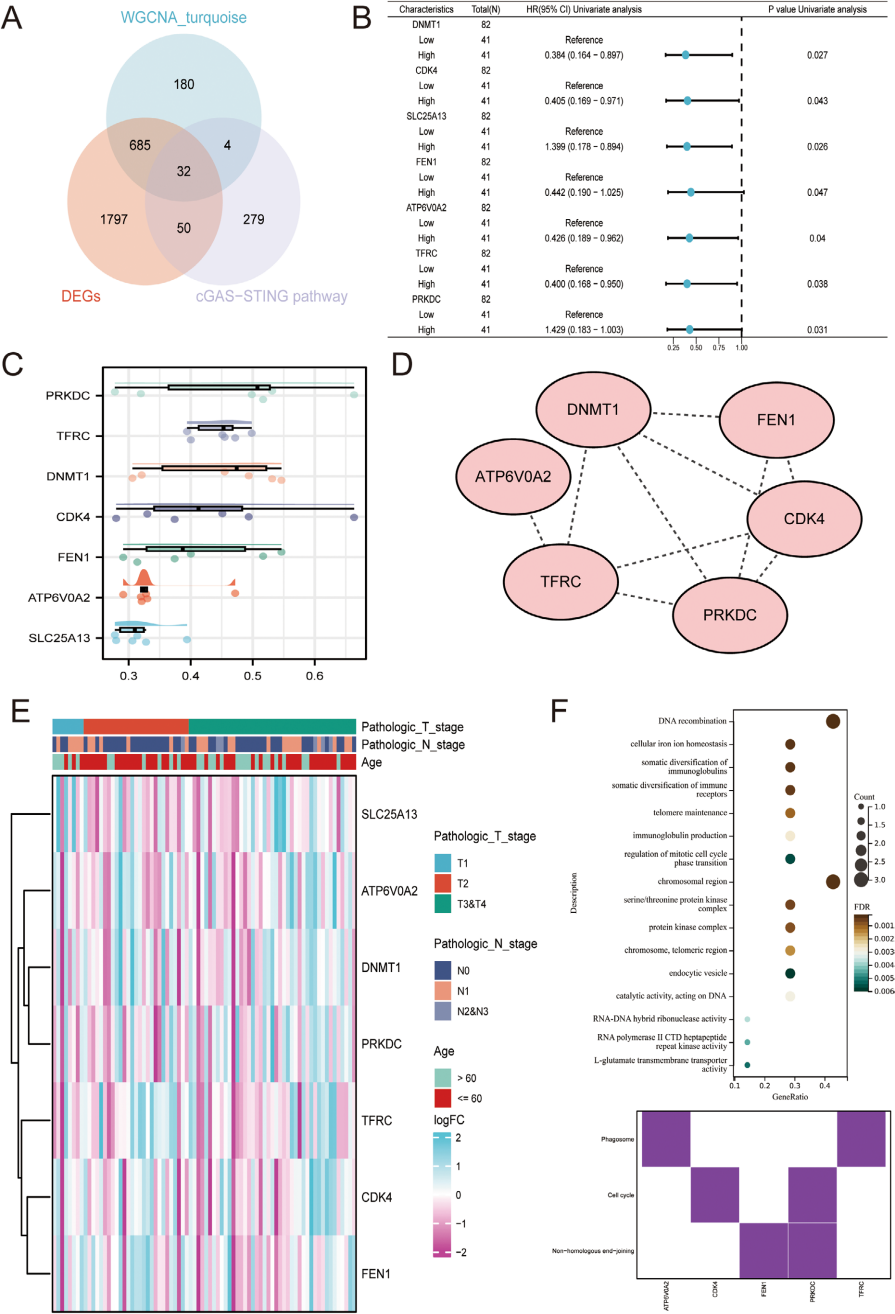
cGAS‐STING pathway associated prognostic indicators identification. (A) Venn diagram illustration of cGAS‐STING pathway‐related DEGs. (B) Univariate Cox regression analysis. (C) Friend analysis. (D) PPI network analysis. (E) Expression pattern analysis. (F) KEGG and GO enrichment analysis. Data with threshold of *p* < 0.05 was considered as significant.

By integrating the Limma and WGCNA analysis with cGAS‐STING pathway gene list, we acquired 32 DEGs (Figure 3A). Next, univariate cox regression was performed to identify prognostic indicators among these 32 DEGs (Figure 3B). The results indicated that there are 7 cGAS‐STING pathway‐associated indicators in the TCGA‐ESCC cohort. In addition, Friend and PPI network analysis was conducted for identifying the important indicators (Figure 3C,D). Indeed, the expression patterns of these 7 indicators with clinical parameters in the TCGA‐ESCC cohort and their corresponding molecular functions were also assessed (Figure 3E,F).

### 3.3 cGAS‐STING Associated Consensus Subgroups Recognition for ESCC Patients

**FIGURE 4:**
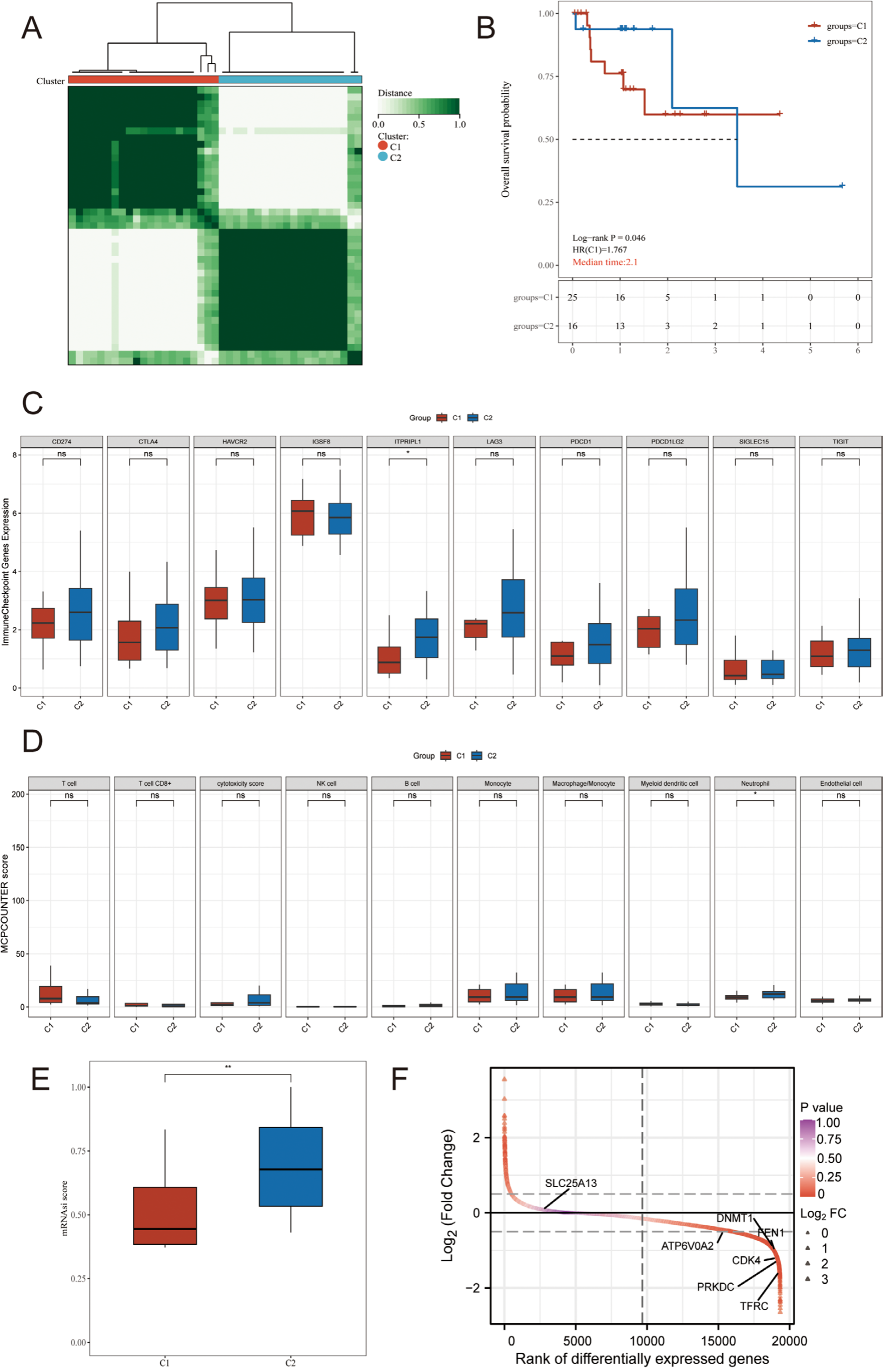
cGAS‐STING pathway associated subgroups identification. (A) Consensus heatmap. (B) KM analysis. (C) Immune checkpoint expression patterns. (D) The proportion of immune cells between C1 and C2. (E) Tumor stemness analysis. (F) Expression patterns of 7 cGAS‐STING associated indicators. Data with threshold of *p* < 0.05 and was considered as significant.

In TCGA‐ESCC cohort, based on these 7 indicators, we performed consensus clustering for identification of cGAS‐STING pathway‐associated subgroups. The results illustrated that cGAS‐STING‐associated 7 indicator can divide ESCC patients into 2 clearly defined subgroups when k = 2 (Figures 4A and S2A–C). C1 ESCC subgroup illustrated the unfavorable prognosis (Figure 4B). Indeed, the expression patterns of immune checkpoint, immune cell proportions, tumor stemness, and expression patterns of 7 indicators were also assessed (Figure 4C–F). Besides, the clinical parameters between C1 and C2 were also compared (Figure S2D).

### 3.4 cGAS‐STING Associated Prognostic Model Construction

**FIGURE 5:**
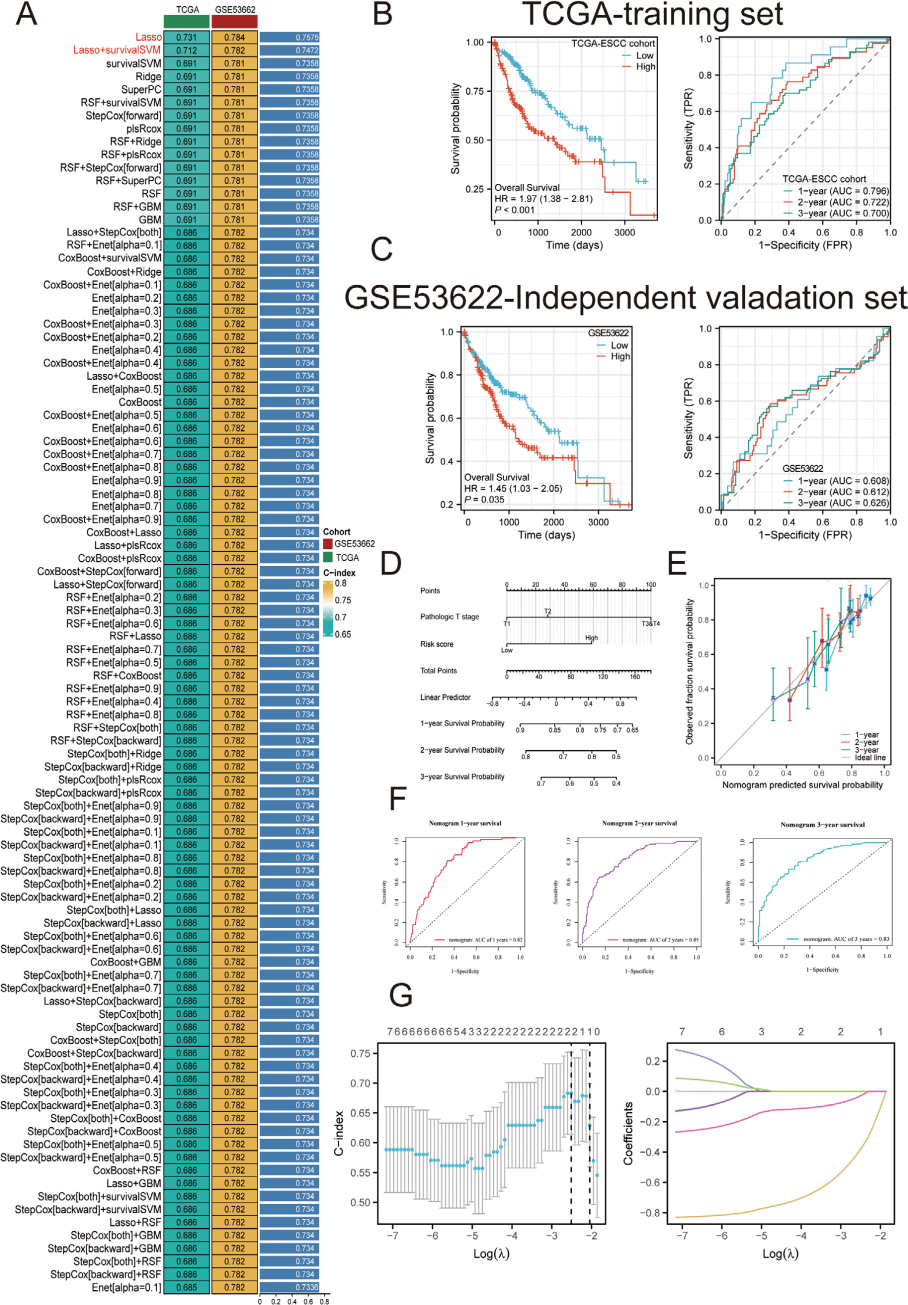
cGAS‐STING pathway associated prognostic model. (A) A 10‐Fold cross‐validation framework enables C‐Index calculation for 101 distinct prediction models. (B) Distinguishing high cGAS‐STING pathway and low cGAS‐STING pathway samples in the TCGA‐ESCC cohort. (C) Distinguishing high cGAS‐STING pathway and low cGAS‐STING pathway samples in the GSE53662 (C–F) cGAS‐STING associated‐prognostic model accuracy and efficacy evaluation in TCGA‐ESCC cohort. (G) Lasso‐cox regression analysis for identified cGAS‐STING associated‐prognostic hub genes. Data with a threshold of *p* < 0.05 was considered significant.

Utilizing the TCGA‐ESCC dataset for our training, we developed a total of 101 models based on LOOCV and determined that the Lasso regression and Lasso regression with SurvivalSVM combination emerged as the most effective methodology, yielding the highest concordance index (C‐index) as illustrated in Figure 4A. Indeed, we selected Lasso regression with SurvivalSVM for construction of prognostic model. This analytical framework proficiently categorized patients into high‐risk and low‐risk cohorts within both the TCGA‐ESCC cohort and GSE53662 datasets, as shown in Figure 4B,C. To verify model independent prognostic efficacy in TCGA‐ESCC cohort, results from nomogram and calibration analysis demonstrated that cGAS‐STING‐related model served as a significant prognostic factor (Figure 5D–F). Lasso‐cox regression analysis confirmed that SLC25A13 and PRKDC can be considered as 2 hub prognostic factors involved in ESCC pathogenesis (Figure 5G).

### 3.5 SLC25A13 and PRKDC Immune and Molecular Feature Analysis

**FIGURE 6:**
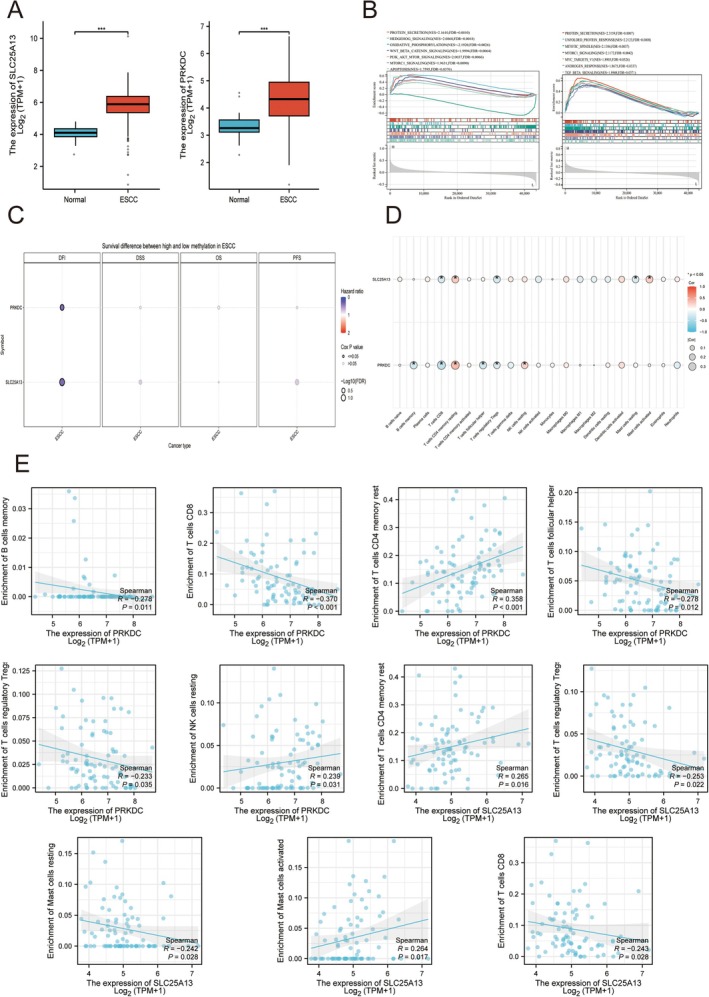
Molecular and immune features of PRKDC and SLC25A13 in ESCC patients. (A) Expression value analysis of PRKDC and SLC25A13 in TCGA‐ESCC cohort compared to normal controls. (B) Single‐gene GSEA analysis enrichment. (C) Methylation analysis. (D) Immune infiltration analysis. (E) The proportion of immune cells with targeted gene expression. Data with a threshold of *p* < 0.05 was considered significant.

In TCGA‐ESCC cohort studies, we discovered that SLC25A13 and PRKDC were positively expressed in ESCC samples compared to normal samples (Figure 6A). The molecular functions of SLC25A13 and PRKDC were also enriched in TCGA‐ESCC cohort (Figure 6B). The DNA methylation and methylation level with survival status of ESCC patients were analyzed (Figure 6C). In addition, The correlation of PRKDC and SLC25A13 with immune cell proportions was evaluated and discovered that these 2 targeted genes were associated with various immune cell types (Figure 6E).

### 3.6 Performance of SLC25A13 and PRKDC at Single‐Cell Level of ESCC Patients

**FIGURE 7:**
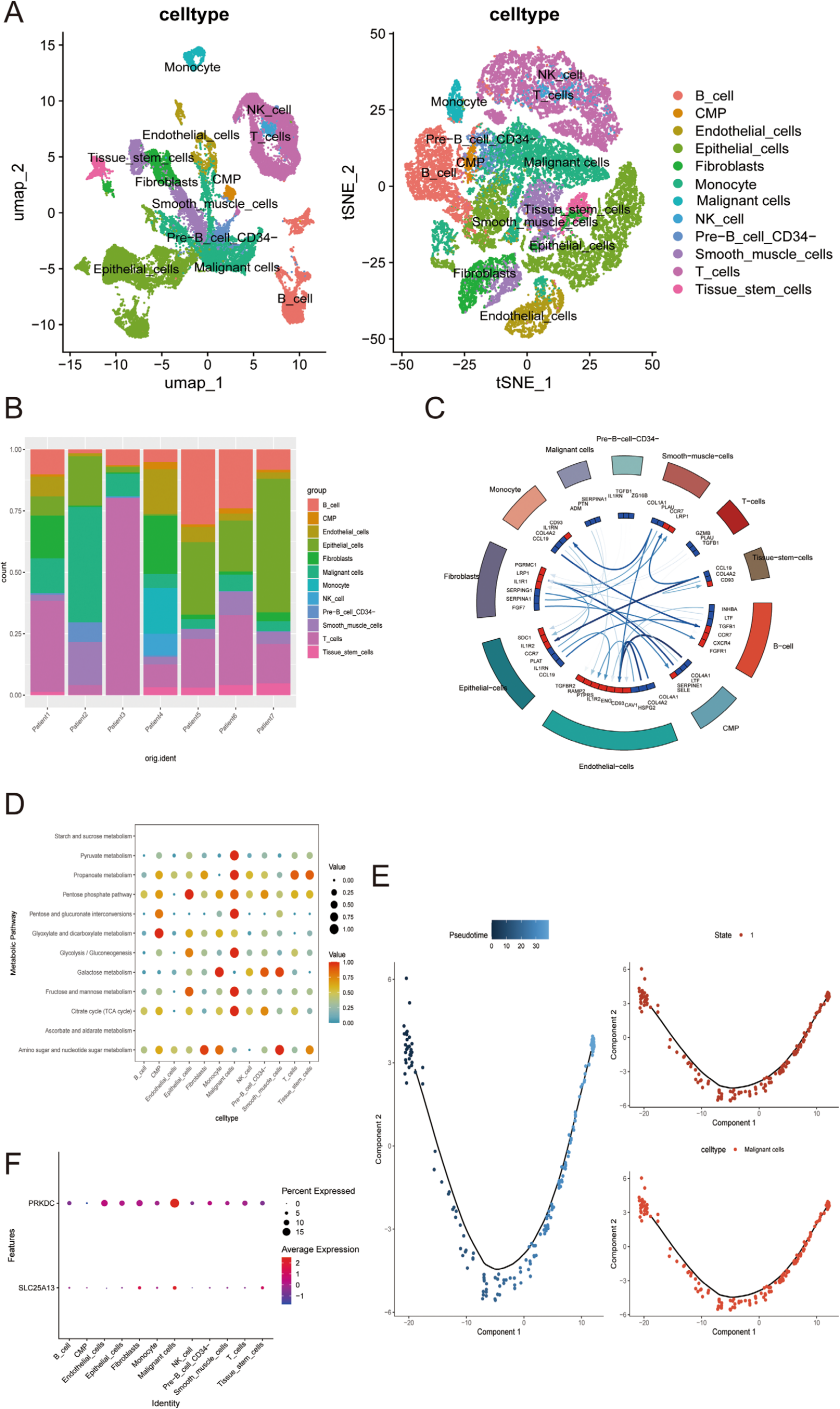
PRKDC and SLC25A13 in ESCC single‐cell level. (A) t‐SNE and UMAP plots of annotated cell types in GSE188900. (B) The cell proportion of annotated cell types in GSE188900. (C) Celltalk analysis of annotated cell types in GSE188900. (D) Energy metabolism patterns among annotated cell types in GSE188900. (E) Differentiation patterns of malignant cells. (F) PRKDC and SLC25A13 distribution in annotated cell types in GSE188900. Data with threshold of *p* < 0.05 was considered as significant.

In ESCC single cell patient profile (GSE188900), the dataset underwent normalization and QC measures (Figure S3A–C). To facilitate a more precise identification of cell types, we classified the cells into 34 distinct clusters based on the expression profiles of well‐established markers (Figure S3D–G). Subsequent to this annotation process, we identified the presence of 12 unique cell types (Figure 7A,B). Moreover, we also assessed cell communication dynamics with ligand‐receptor mechanisms (Figure 7C). Energy metabolism patterns among these 12 unique cell types were also estimated (Figure 7D). Furthermore, we also identified PRKDC and SLC25A13 distributions among these 12 cell types, which were mainly expressed in malignant cells (Figure 7F). The differentiation pattern of malignant cells in ESCC patients at single‐cell was also assessed (Figure 7E).

### 3.7 SLC25A13 and PRKDC Targeted Therapeutic Framework Identification for ESCC Patients

**FIGURE 8:**
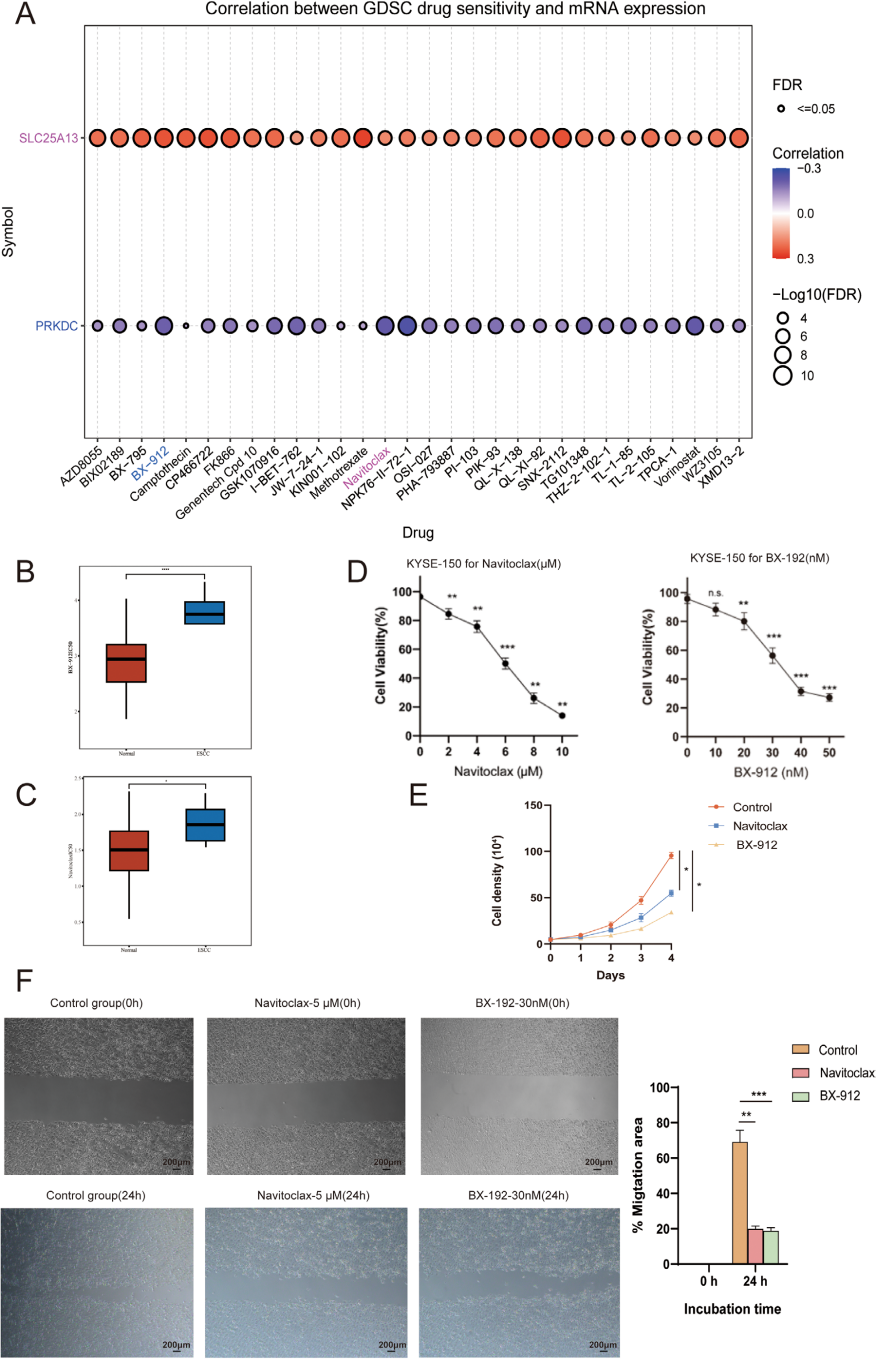
PRKDC and SLC25A13‐oriented drug framework identification for ESCC patients. (A) GSCA enrichment analysis for identification of sensitive drugs targeting PRKDC and SLC25A13. (B,C) Sensitivity analysis of drugs for ESCC patients. (D) CCK‐8 assays for optimum concentration of Navitoclax and BX‐912 in KYSE150 cell lines. (E) CCK‐8 assay for evaluation of Navitoclax and BX‐912 in KYSE150 cell line growth. (F) Wound‐healing assays following Navitoclax and BX‐912 treatment in KYSE150 cells. Data was presented as mean ± SD, *p < 0.05, **p < 0.01, ***p < 0.001. Scale bar = 200 μm.

For drug‐target sensitivity analysis, we found that BX‐912 and Navitoclax were 2 PRKDC and SLC25A13‐oriented drugs (Figure 8A). Indeed, these 2 drugs also illustrated sensitive to ESCC patients (Figure 8B). Molecular docking results indicated that BX‐912 and Navitoclax were 2 optimal drugs with high binding affinity (Figure S4A,B). To explore the optimal concentration of Navitoclax and BX‐912. CCK8 and wound healing assays were performed in KYSE150 cells (Figure 8C). Navitoclax was diluted to 2, 4, 6, 8, and 10 μM, BX‐912 was diluted to 10, 20 30, 40, and 50 nM, and was added to KYSE150, respectively, we found the inhibition of cell survival rate was enhanced with the increase of Navitoclax and BX‐912 concentration in KYSE150 (Figure 8C). When the concentrations reached 6.040 μM of Navitoclax (Figure 1a) and 33.88 nM (Figure 8C). CCK8 and wound healing assays indicated that BX‐912 and Navitoclax can significantly inhibit ESCC cancer cell growth., highlighting their ESCC therapeutic potential (Figure 8D,E).

### 3.8 The Association of SLC25A13 and PRKDC With ESCC Progression

**FIGURE 9:**
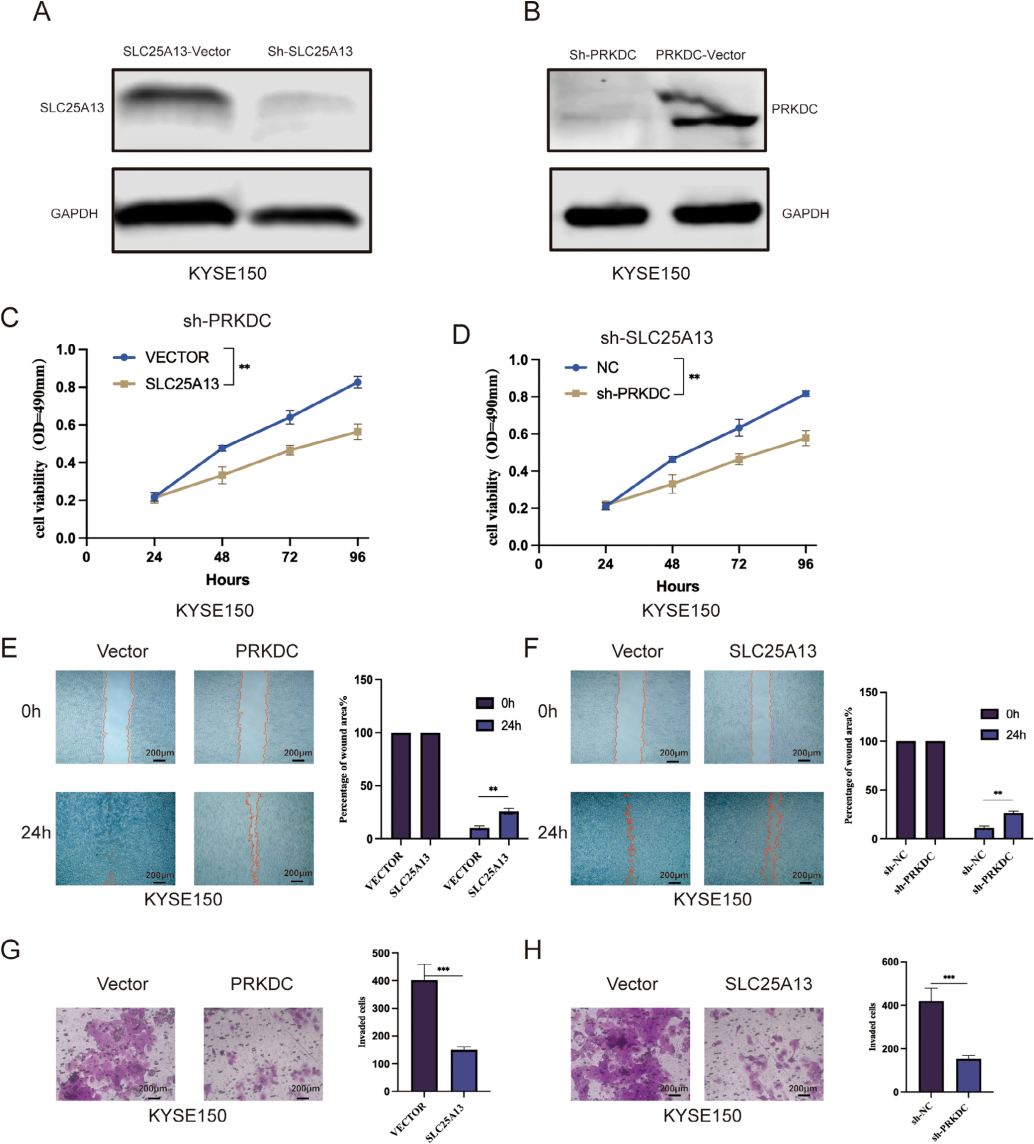
Functional validation of SLC25A13 and PRKDC in ESCC cells. (A) WB analysis confirmed efficient suppression of SLC25A13 expression in cells. (B) WB analysis confirmed efficient knockout of PRKDC in KYSE150 cells. (C) Cell proliferation assays following SLC25A13 and PRKDC knockdown in KYSE150 cells. (D) Wound‐healing assays following SLC25A13 and PRKDC knockdown in KYSE150 cells. (E) Transwell invasion assays following SLC25A13 and PRKDC knockdown in KYSE150 cells. Data was presented as mean ± SD, *p < 0.05, **p < 0.01, ***p < 0.001. Scale bar = 200 μm.

In order to further substantiate the functional involvement of SLC25A13 and PRKDC in ESCC, we conducted a series of in vitro experiments utilizing the KYSE150 cell line. The results of Western blot analysis demonstrated a successful suppression of SLC25A13 and PRKDC expression (Figure 9A,B). Additionally, cell proliferation, colon, and Transwell assays indicated that the suppression of SLC25A13 and PRKDC expression in KYSE150 can inhibit the proliferation, migration, and invasion of the ESCC cell line, thereby endorsing their function as a potential oncogenic driver (Figure 9C–H).

### 3.9 The Estimation of SLC25A13 and PRKDC With cGAS‐STING Pathway and ESCC Pathogenesis

**FIGURE 10:**
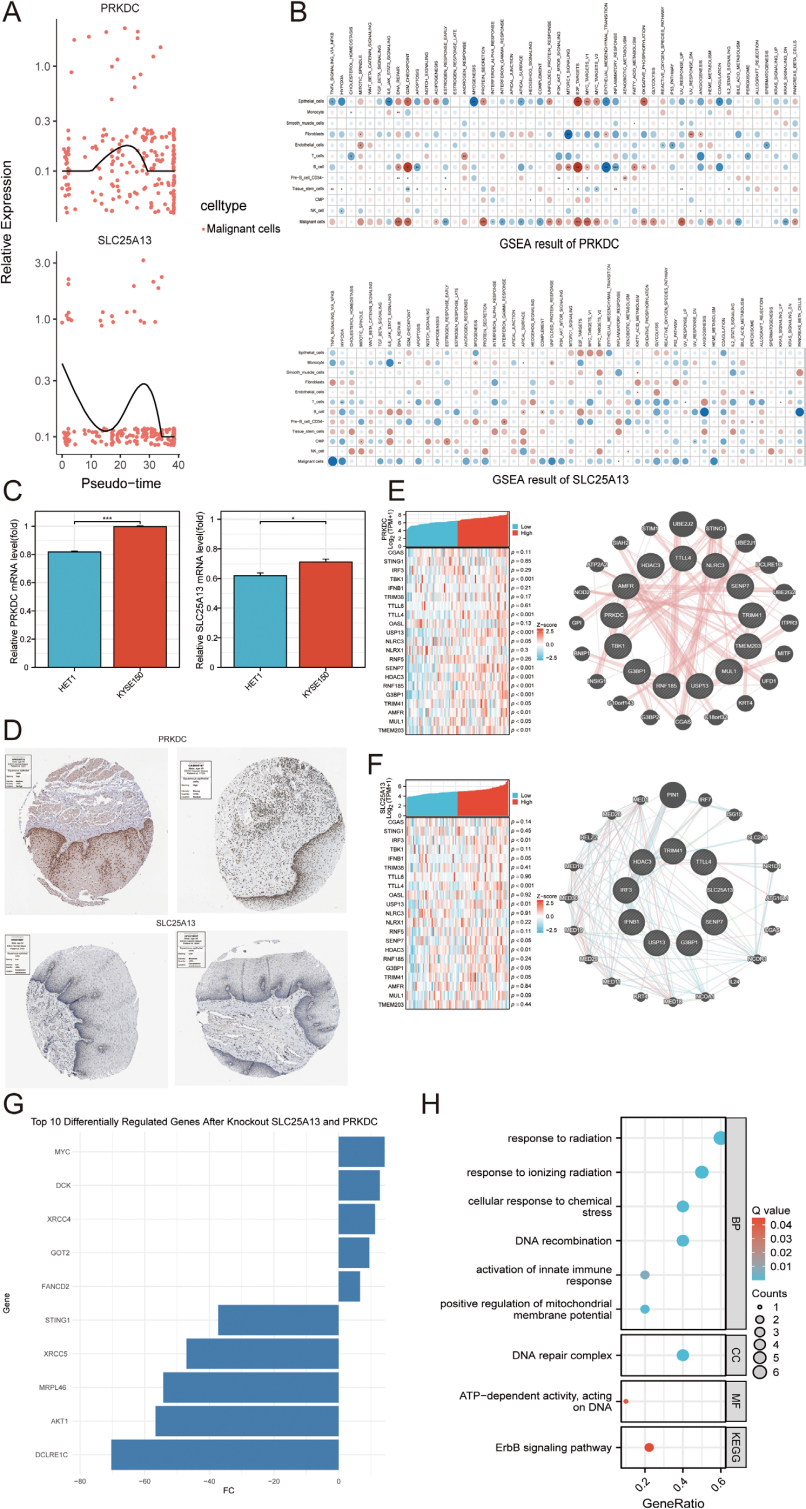
Estimation of SLC25A13 and PRKDC with cGAS‐STING pathway and ESCC pathogenesis. (A) Pseudotime trajectory of SLC25A13 and PRKDC expression in malignant cells. (B) scGSEA analysis of SLC25A13 and PRKDC. (C) Expression estimation of SLC25A13 and PRKDC in KYSE150 cell line compared to HET1 via q‐RT‐PCR. (D) Expression estimation of SLC25A13 and PRKDC at a histological level. (E) Implications of PRKDC with cGAS‐STING pathway. (F) Implications of SLC2513 with cGAS‐STING pathway. (G,H) KO of SLC25A13 and PRKDC in malignant cells. Data was presented as mean ± SD, *p < 0.05, **p < 0.01, ***p < 0.001.

In malignant cells, the expression of SLC25A13 and PRKDC was high in the termination of malignant cell differentiation (Figure 10A). Next, the molecular functions of SLC25A13 and PRKDC in 12 cell types were estimated for deciphering the molecular heterogeneity of SLC25A13 and PRKDC in ESCC pathogenesis (Figure 10B). In mRNA and pathological levels, we discovered that PRKDC and SLC25A13 was expressed higher in ESCC group compared to normal controls (Figure 10C,D). Furthermore, we found that PRKDC was co‐expressed with cGAS‐STING pathway modulators, such as TBK1, TTL4, USP13, NLRC3, SENP7, HDAC3, RNF185, G3BP1, TRIM41, AMFR MUL1 and TMEM203 (Figure 10E). GeneMANIA analysis also can reveal that PRKDC can potentially regulate cGAS‐STING pathway via G3BP1 (Figure 10E). Besides, SLC25A13 was co‐expressed with cGAS‐STING pathway modulators, such as IRF3, IFNB1, TTLL4, USP13,SENP7, HDAC3, G3BP1 and TRIM41 (Figure 10E). GeneMANIA analysis also can reveal that SLC25A13 can potentially regulate cGAS‐STING pathway via TRIM41 (Figure 10F). Furthermore, in AI‐empowered malignant virtual cells, after KO of SLC25A13 and PRKDC, we discovered that the down‐regulated expression of STING, indicating that the correlation between SLC25A13 and PRKDC, and cGAS‐STING pathway (Figure 10G). Molecular functions of Top10 DEGs after SLC25A13 and PRKDC KO in malignant virtual cells were analyzed (Figure 10H).

## 4 Discussion and Conclusion

ESCC remains a significant global health challenge, with limited therapeutic options and poor prognostic outcomes [32]. Current treatment strategies often fail to account for the heterogeneity of the disease, leading to suboptimal patient management and outcomes [33]. This underscores the necessity of identifying novel biomarkers and therapeutic targets that can enhance stratification and treatment efficacy in ESCC. In this study, we focused on the innate immune regulator, cGAS‐STING pathway, which has emerged as a critical player in immune response and tumor microenvironment [34]. By integrating AI and multi‐omic pipelines, we demonstrated that this pathway not only aids in subgroup stratification but also plays a pivotal role in prognostic model construction for ESCC. Furthermore, our findings identified PRKDC and SLC25A13 as hub genes associated with ESCC pathogenesis, highlighting their potential as therapeutic targets.

PRKDC (Protein Kinase, DNA‐Activated, Catalytic Subunit) plays a crucial role in the DNA damage response and repair mechanisms, particularly in the non‐homologous end joining (NHEJ) pathway, which is vital for maintaining genomic stability [35]. Its dysregulation has been implicated in various cancers, contributing to tumor progression by facilitating the survival of malignant cells under genotoxic stress [36]. In addition, PRKDC‐induced DNA damage can lead to the activation of the cGAS‐STING pathway and glioblastoma tumor‐promoting inflammation [37]. Besides, PRKDC was significantly amplified for increased DNA repair pathway to enhance radio‐sensitivity of ESCC [38]. However, there are limitations in research targeting PRKDC for regulating the cGAS‐STING pathway and ESCC progression. Novelty, in our study, we preliminarily identified that PRKDC can potentially regulate the cGAS‐STING pathway via G3BP1, which may potentially contribute to ESCC progression. SLC25A13 (Solute Carrier Family 25 Member 13) is involved in mitochondrial transport processes, particularly in the transport of aspartate and glutamate across the mitochondrial membrane [39]. Report has been implicated SLC25A13 was associated with unfavorable clinical outcomes of ESCC patients [40]. This gene has been linked to metabolic regulation and apoptosis, both of which are critical in cancer biology [41]. SLC25A13 plays a crucial role in the immune surveillance of cancer [42]. However, the mechanisms of SLC25A13 in ESCC progression and cGAS‐STING modulation have not yet been further elucidated. In our study, we illustrated that SLC25A13 can potentially regulate the cGAS‐STING pathway via TRIM41 for involvement in ESCC pathogenesis. Besides, in virtual malignant cells, KO of SLC25A13 and PRKDC can significantly affect STING expression, highlighting their potential on regulating the cGAS‐STING pathway for involving ESCC progression. BX‐912 and Navitoclax have been proved to be potential drugs for the treatment [43, 44]. In our study, we firstly proved that these 2 drugs can be considered as a therapeutic framework for ESCC patients by targeting SLC25A13 and PRKDC.

Overall, we first constructed cGAS‐STING pathway‐associated model and molecular subgroups for ESCC patients. *In silico* and in vitro studies proved the dominant role of SLC25A13 and PRKDC in ESCC progression via cGAS‐STING pathway. In addition, BX‐912 and Navitoclax can be considered as drug screening frameworks for the treatment of ESCC targeting SLC25A13 and PRKDC. However, there are limitations of our study. First, the mechanism of SLC25A13 and PRKDC in regulating cGAS‐STING pathway for modulating ESCC progression should be validated in pre‐clinical study. Besides, therapeutic efficacy and safety of BX‐912 and Navitoclax targeting SLC25A13 and PRKDC should be validated in animal studies and multi‐center studies.

## Author Contributions

Chunyang Zhou, Xiaoli Liu, and Zijian Wang designed the study and performed data analysis. Chunyang Zhou, Xiaoli Liu, and Zijian Wang conducted machine learning modeling and validation. Chunyang Zhou and Xiaoli Liu carried out single‐cell and drug discovery analyses. Chunyang Zhou and Xiaoli Liu conducted experimental validation. Chunyang Zhou prepared the figures. Tao Yang supervised the project. All authors contributed to writing and reviewing the manuscript.

## Funding

The authors have nothing to report.

## Conflicts of Interest

The authors declare no conflicts of interest.

## Supporting information


**Data S1:** Supporting Information Figures.

## Data Availability

Data supporting the findings of this study is publicly available. TCGA‐ESCC data can be accessed at https://portal.gdc.cancer.gov/. GSE38129, GSE53662, and GSE188900 were scored from https://www.ncbi.nlm.nih.gov/geo/. ESCC histological cancer and para‐cancer samples can be found in HPA database https://www.proteinatlas.org/. IHC samples of ESCC patients can be found in https://www.proteinatlas.org/.
