# Supplementary material for: Identification and Validation of cGAS‐STING Pathway‐Associated Predictive and Therapeutic Models for Esophageal Squamous Cell Cancer Patients via Artificial Intelligence and Multi‐Omics
Source: Cancer Med. 2026 Feb 23;15(3):e71645. doi: 10.1002/cam4.71645 (PMC12929672; doi:10.1002/cam4.71645)
Supplement: Supplementary file 1 — Data S1: Supporting Information Figures. [file CAM4-15-e71645-s001.docx]

**
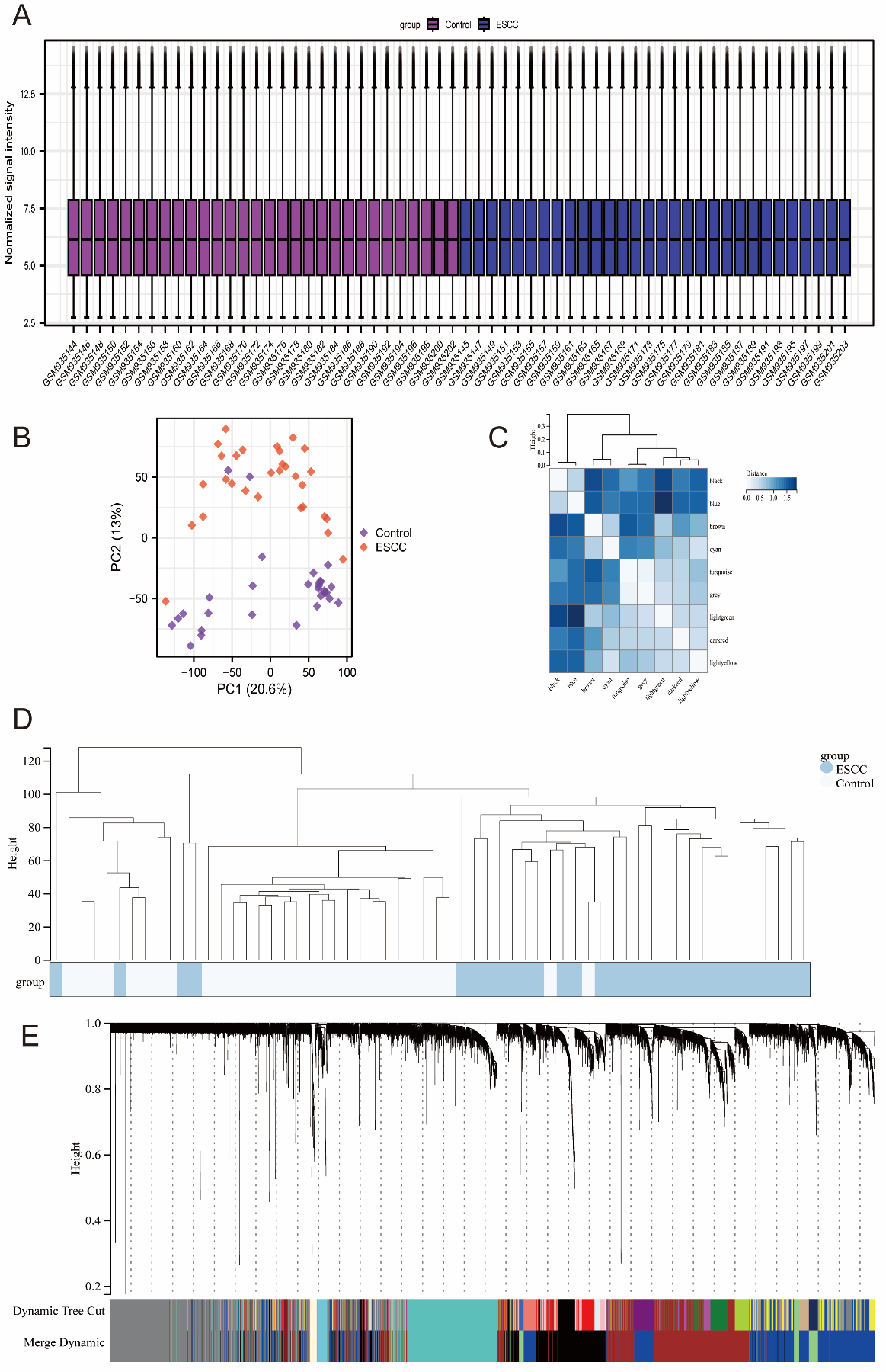
**

**Figure S1: Limma and WGCNA frameworks in** **GSE38129. (A)** Box plot illustration of GSE38129 normalization. **(B)** PCA plot illustration separation. **(C)** Correlation among various modules of WGCNA analysis. **(D)** Clustering tree of WGCNA analysis among samples. **(E)** Clustering tree of expression module via WGCNA analysis.


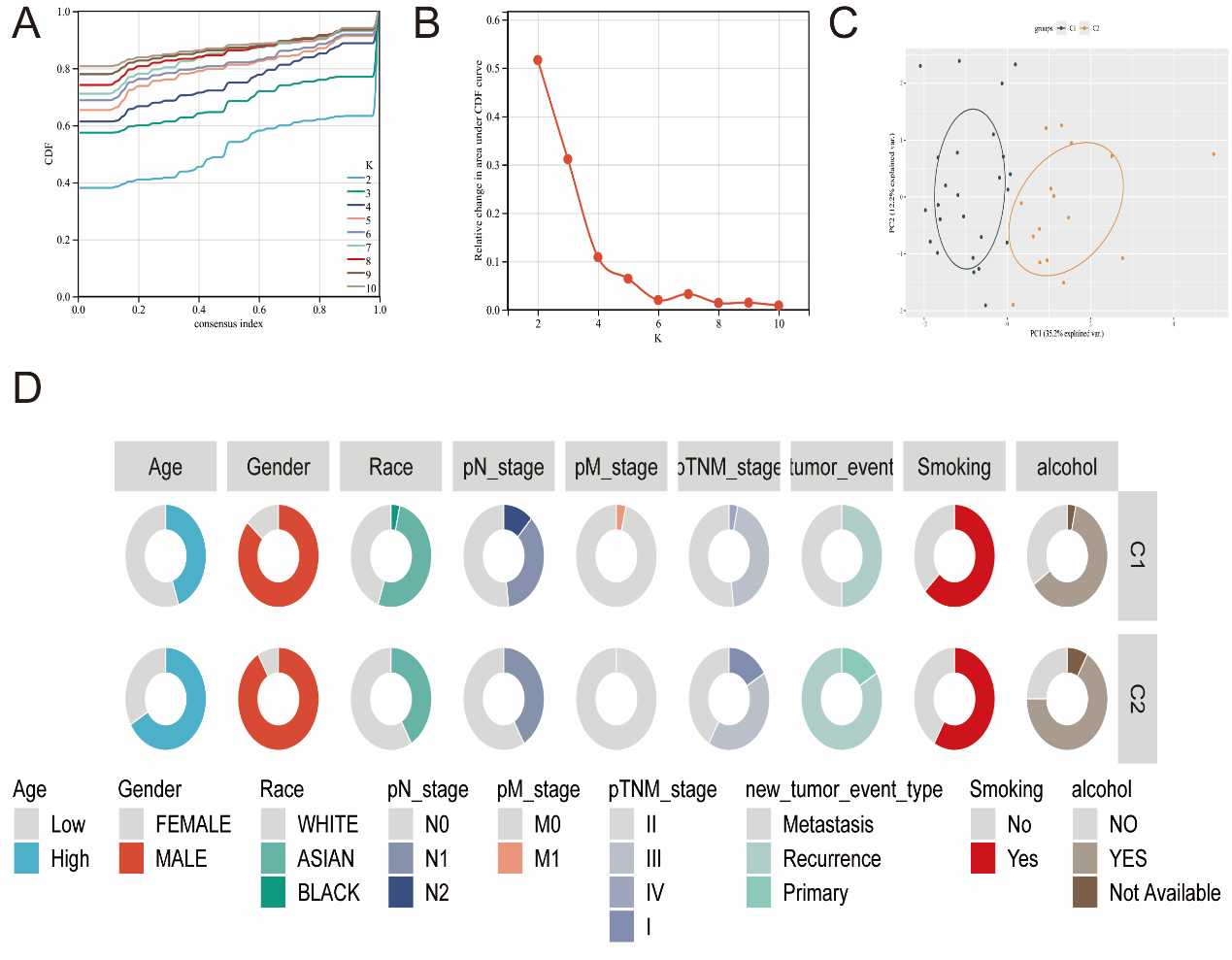


**Figure S2: Consensus clustering information. (A)** Consensus index illustration. **(B)** CDF curve illustration.**(C)** PCA plot illustration of clustering results. **(D)** Clinical parameters difference among subgroups.


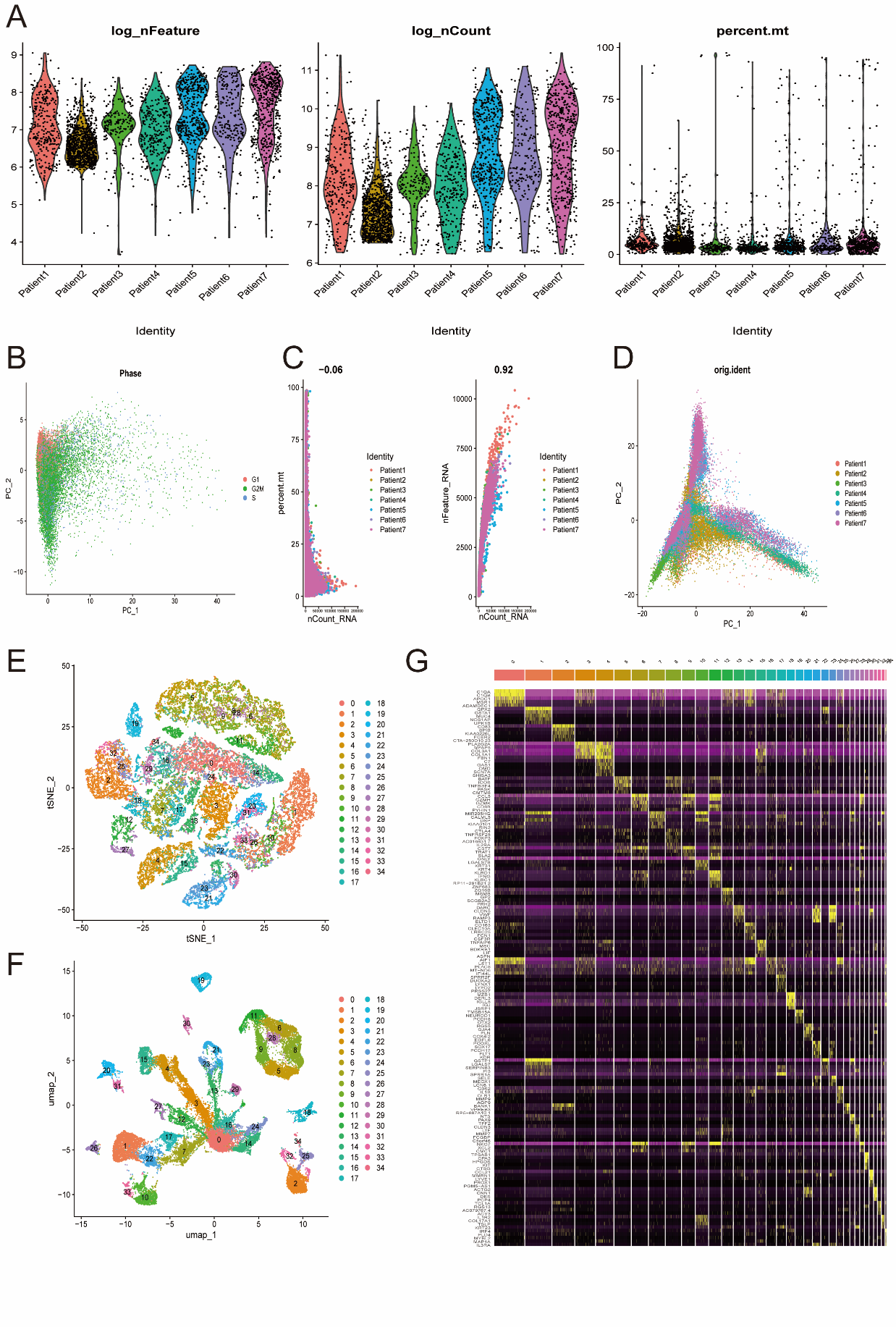


**Figure S3: Pre-processing of single-cell data. (A-C)** QC metrics of single-cell data across samples. **(D)** PCA analysis of cells based on cell cycle phase (G1, S, G2M). **(E-F)** Dimensionality reduction using t-SNE and UMAP. **(G)** Dimensionality reduction markers.


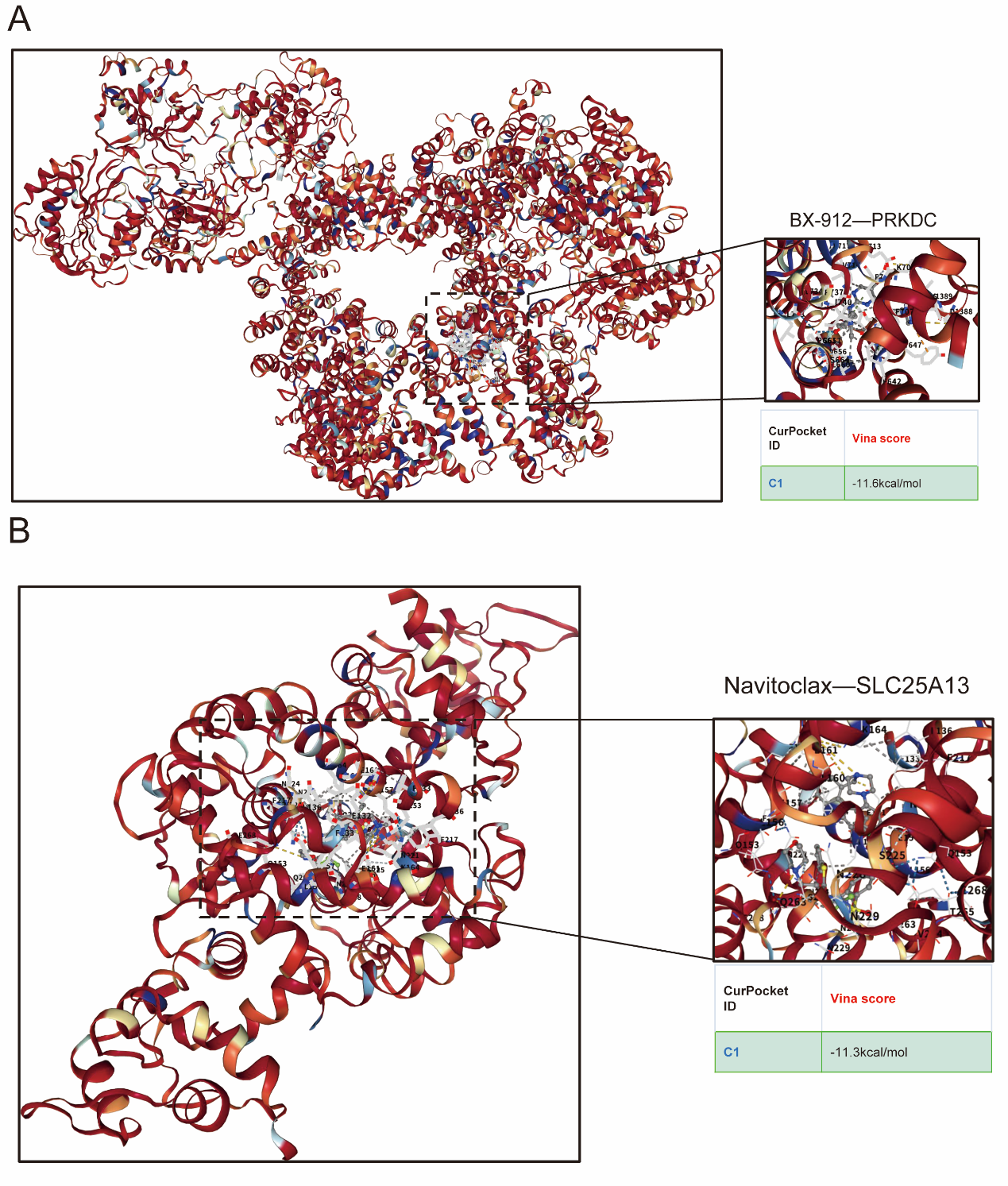


**Figure S4: Molecular docking analysis. (A)** Molecular docking analysis between BX-912 and PRKDC. **(B)** Molecular docking analysis between Navitoclax andSLC25A13.
